# Identification of multidimensional Boolean patterns in microbial communities

**DOI:** 10.1186/s40168-020-00853-6

**Published:** 2020-09-11

**Authors:** George Golovko, Khanipov Kamil, Levent Albayrak, Anna M. Nia, Renato Salomon Arroyo Duarte, Sergei Chumakov, Yuriy Fofanov

**Affiliations:** 1 Department of Pharmacology and Toxicology, University of Texas Medical Branch–Galveston, Galveston, TX 77555-0144 USA; 2 Sealy Center for Structural Biology and Molecular Biophysics, University of Texas Medical Branch–Galveston, Galveston, TX 77555-0144 USA; 3 Department of Molecular Biophysics, University of Texas Medical Branch–Galveston, Galveston, TX 77555-0144 USA; 4 Department of Physics, University of Guadalajara, Revolucion, 1500 Guadalajara, Jalisco Mexico

**Keywords:** Microbiome, Multidimensional Boolean patterns, Microbial communities, Co-exclusion, Co-presence, Pattern-specific score

## Abstract

**Background:**

Identification of complex multidimensional interaction patterns within microbial communities is the key to understand, modulate, and design beneficial microbiomes. Every community has members that fulfill an essential function affecting multiple other community members through secondary metabolism. Since microbial community members are often simultaneously involved in multiple relations, not all interaction patterns for such microorganisms are expected to exhibit a visually uninterrupted pattern. As a result, such relations cannot be detected using traditional correlation, mutual information, principal coordinate analysis, or covariation-based network inference approaches.

**Results:**

We present a novel pattern-specific method to quantify the strength and estimate the statistical significance of two-dimensional *co-presence*, *co-exclusion*, and *one-way relation* patterns between abundance profiles of two organisms as well as extend this approach to allow search and visualize three-, four-, and higher dimensional patterns. The proposed approach has been tested using 2380 microbiome samples from the Human Microbiome Project resulting in body site-specific networks of statistically significant 2D patterns as well as revealed the presence of 3D patterns in the Human Microbiome Project data.

**Conclusions:**

The presented study suggested that search for Boolean patterns in the microbial abundance data needs to be pattern specific. The reported presence of multidimensional patterns (which cannot be reduced to a combination of two-dimensional patterns) suggests that multidimensional (multi-organism) relations may play important roles in the organization of microbial communities, and their detection (and appropriate visualization) may lead to a deeper understanding of the organization and dynamics of microbial communities.

Video Abstract

## Background

Identification of complex multidimensional patterns of abundances/appearances among members of microbial communities (MC) is the key to understand, control, and (in the future) design beneficial microbial communities as well as guide microbial transplantation and personalize antimicrobial and probiotic treatments. Since members of microbial communities can be simultaneously involved in multiple relations that altogether will determine their abundance, not all significant relations between organisms are expected to be manifested as visually uninterrupted patterns and be detected using traditional correlation, mutual information, principal coordinate analysis, or covariation-based approaches. They, however, might be identified and described using Boolean two-, three-, and higher dimensional patterns.

### Non-continuous multidimensional patterns

To a certain extent, complex relations between microorganisms within microbial communities (MC) can be recovered by observing their abundances as well as monitoring how they change in response to internal and external perturbations/variables [1]. While initial microbiome characterization studies have been focused on detection of particular organisms under different conditions (healthy vs diseased state), recent studies employ pairwise microbial interaction network analysis to provide a deeper understanding of interactions in MC [2–5]. Traditional methods are generally used to recover pairwise relations between microorganisms in MC which include mutual information-based approaches such as MIC [6], Pearson’s or Spearman’s correlation [7, 8], and covariation. Several computational tools utilizing mutual information, correlation, and covariation techniques have become an essential part of advanced analysis to identify interaction patterns in microbial communities [9–13]. Tools like SparCC [14], developed to infer correlation networks from compositional data, and CoNet [15], which uses an ensemble method to combine information from several different standard comparison metrics, have become widely accepted by the scientific community. While methods based on mutual information (MI), such as MIC [16], are capable of identifying nonlinear and non-continuous pairwise relations [17], they lack the ability to discriminate against intuitively difficult to interpret patterns and can miss some important relationships such as mutual exclusion among microorganisms [18].

**Fig. 1:**
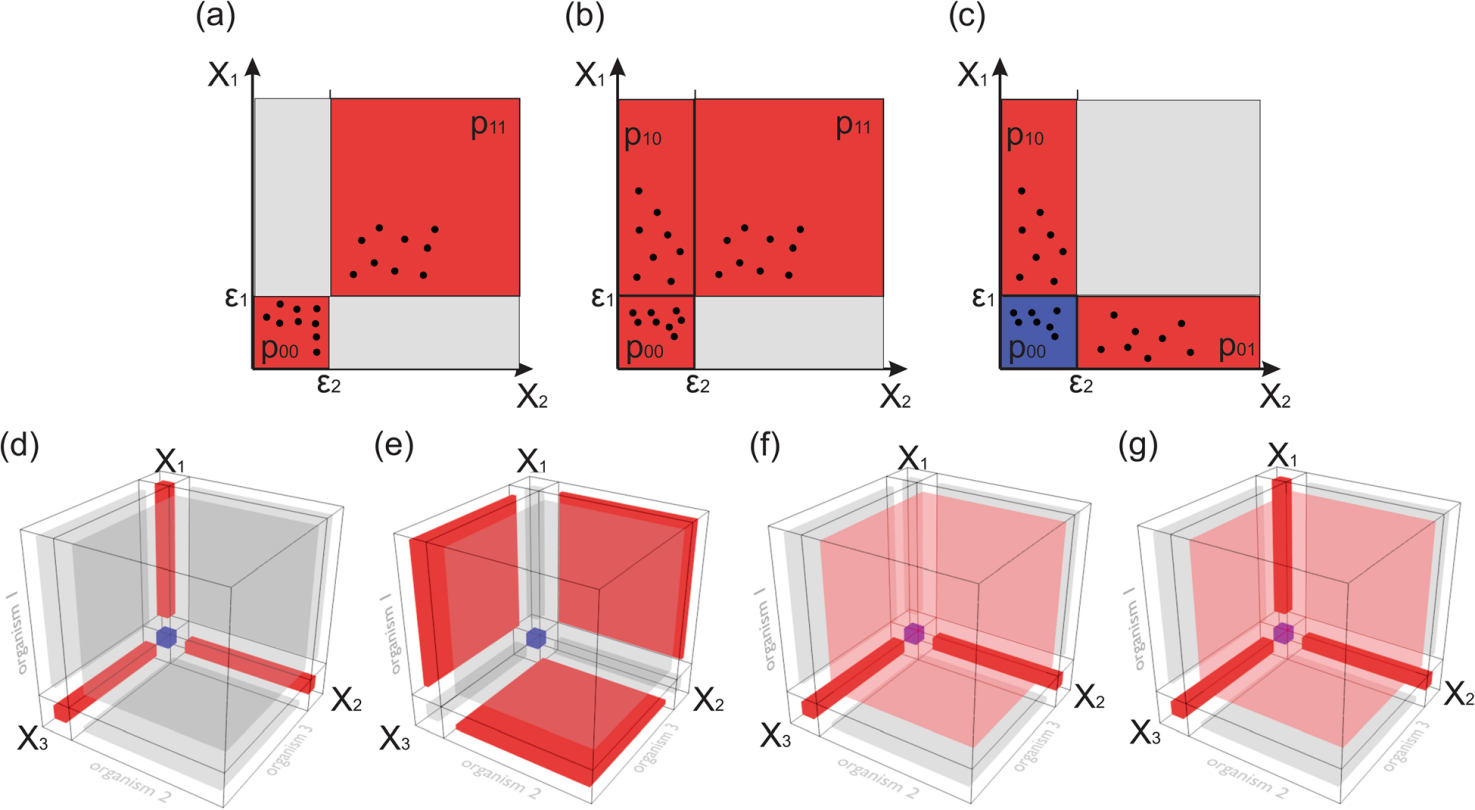
Examples of non-continuous two- and three-dimensional Boolean patterns. Two-dimensional patterns: **a** *co-presence*, **b** *one-way*, and **c** *co-exclusion* patterns. *X*_1_ and *X*_2_ are the abundances of microorganisms; *ε*_1_ and *ε*_2_ represent the presence/absence threshold; *p*_00_, *p*_01_, *p*_10_, and *p*_11_ are the proportion of points (observation) located in each partition. Three-dimensional patterns: **d** type 1 *co-exclusion*, **e** type 2 *co-exclusion*, **f** a pattern when the presence of organism *X*_1_ changed patterns between *X*_2_ and *X*_3_ from *co-presence* to *co-exclusion*, and **g** the case where three organisms can be present only all together on one-by-one. Red color represents quadrants requiring the proportion of observation to exceed the minimum threshold. Red and blue quadrants are areas contributing to the pattern score

Some members of MC can be simultaneously involved in multiple relations which together determine their abundance. For example, in many environmental microbial communities, functions vital to the whole community (e.g., nitrogen fixation) are often performed by a single species [19, 20], so the abundance pattern between these organisms and other members of MC will not be represented by correlation, but are rather expected to exhibit a Boolean pattern. A Boolean *one-way relation* pattern is exhibited when the presence of “dependent” microorganism(s) requires the presence of a “provider,” but not vice versa (Fig. 1b). Similarly, other pairwise relations such as *co-presence* and *co-exclusion* may be represented as non-continuous Boolean patterns (Fig. 1a, c). For pairwise relations, there are a total of 2^4^ possible combinations (2^2*n*^, where *n* = number of variables/organisms), given that there are four Boolean functions (constant function true, negation function, identity function, and constant function false) for every Boolean variable [21]. However, out of 2^4^ possible combinations of the presence/absence profiles between two organisms, only four may be interpreted as possible relations: *co-presence*, *co-exclusion*, and two *one-way relations* (organism 1 needs organism 2 to survive and vice versa). It is also important to keep in mind that if the cooperation of several organisms is required to maintain a single metabolic pathway, their abundances will fit into multidimensional Boolean patterns, such as *multidimensional co-presence* (Fig. 1g).

### Complications of using mutual information as a score for non-continuous patterns

In Boolean patterns, two microorganisms’ abundances can vary without affecting the pattern (e.g., variation in abundance within the same quadrant), and the pattern strength can be defined based on the fraction of observations located in four quadrants of two-dimensional space:  *p*_00_, *p*_01_, *p*_01_, *p*_11_ (Fig. 1a–c). However, it is important to mention that since different roles played by microorganisms in MC may require different minimal abundances. The appropriate calculation of the *p*_*ij*_ will require identification of the microorganism-specific thresholds, so *p*_*ij*_ becomes a function of four variables: *p*_*ij*_(*X*_1_, *X*_2_, *ε*_1_, *ε*_2_), where *X*_1_ and *X*_2_ are the abundance profiles of two microorganisms under consideration and *ε*_1_ and *ε*_2_ are the corresponding presence/absence thresholds.

The most obvious choice to define the strength of a Boolean pattern would be by using a mutual information score (MIS):

$$ \mathrm{MIS}\left({X}_1,{X}_2\right)=\underset{\varepsilon_1,{\varepsilon}_2}{\max}\left(\mathrm{MI}\left({X}_1,{X}_2,{\varepsilon}_1,{\varepsilon}_2\right)\right)=\underset{\varepsilon_1,{\varepsilon}_2}{\max}\left({H}_1\left({X}_1,{\varepsilon}_1\right)+{H}_2\left({X}_2,{\varepsilon}_2\right)-{H}_{12}\left({X}_1,{X}_2,{\varepsilon}_1,{\varepsilon}_2\right)\right)\kern0.5em , $$

where *H*_*i*_(*X*_*i*_, *ε*_*i*_) and *H*_*ij*_(*X*_*i*_, *X*_*j*_, *ε*_*i*_, *ε*_*j*_) correspond to one- and two-dimensional entropies.

**Fig. 2:**
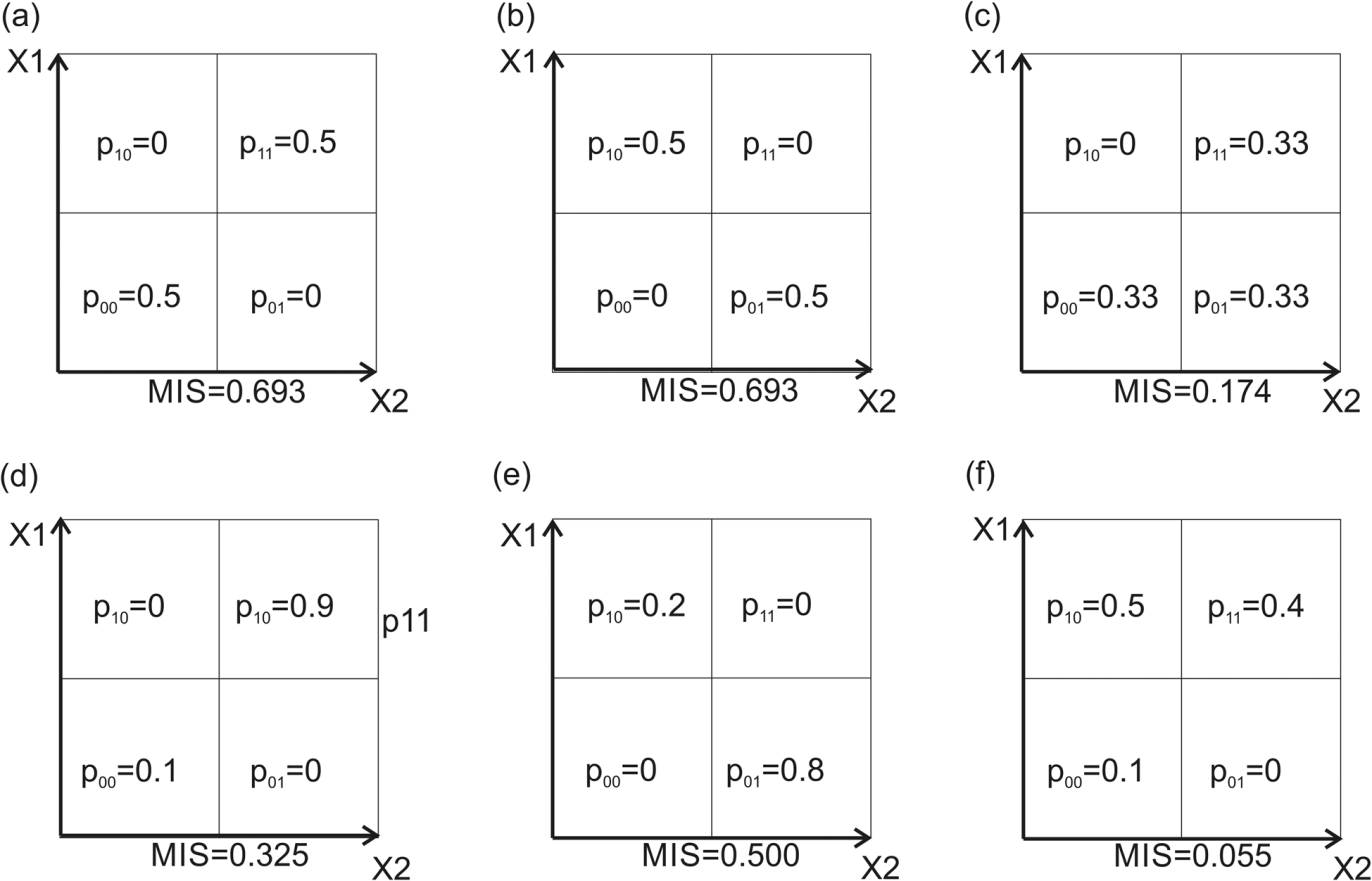
Mutual information score values for different pattern types. MIS values for “ideal” **a** *co-presence*, **b** *co-exclusion*, and **c** *one-way* patterns. Effect of disbalance on **d** *co-presence*, **e** *co-exclusion*, and **f** *one-way* relations on MIS values

The use of mutual information to identify such patterns, however, has several significant disadvantages. The best possible (maximal) MIS value is not the same for different pattern types. For example, while MIS value for “ideal” *co-presence* and *co-exclusion* patterns is 0.693 (Fig. 2a, b), the score for “ideal” *one-way* pattern is only 0.174 (Fig. 2c). Moreover, a small disbalance between fractions of points located in four partitions *p*_00_, …, *p*_11_ can significantly affect the MIS value. For example, while two *co-exclusion* patterns may be intuitively obvious (Fig. 2a, d), a disbalance between *p*_00_ and *p*_11_ can cause a significant drop in the MIS value. A similar observation can be made for *co-exclusion* (Fig. 2b, e). The use of MIS value can be especially misleading in the case of *one-way* relation patterns. An MIS value may be extremely low (0.055) for patterns which can be clearly interpreted as *one-way* relation (Fig. 2f). These observations suggest that the use of MIS to identify non-continuous Boolean patterns may result in missing certain intuitively obvious patterns. The presented work is an attempt to introduce an alternative, pattern-specific approach, to estimate the strength and statistical significance of two- and higher dimensional patterns between members of microbial communities.

## Methods

### Pattern-specific strength score

The basic idea of the proposed approach is to estimate the pattern score by counting the fraction of observations belonging to the pattern under investigation. Assuming that *p*_00_ + *p*_01_ + *p*_10_ + *p*_11_ = 1 and the presence/absence threshold can be microorganism specific, the strength of each pattern can be defined as the following:

$$ {S}_{\mathrm{co}-\mathrm{presence}}=\underset{\varepsilon_1,{\varepsilon}_2}{\max}\left({p}_{00}+{p}_{11}\right); $$

$$ {S}_{\mathrm{co}-\mathrm{exclusion}}=\underset{\varepsilon_1,{\varepsilon}_2}{\max }\ \left({p}_{00}+{p}_{10}+{p}_{01}\right); $$

${S}_{\mathrm{one}-\mathrm{way}}=\underset{\varepsilon_1,{\varepsilon}_2}{\max}\left({p}_{00}+{p}_{01}+{p}_{11}\right)$; (where *X*_2_ depends on *X*_1_)

${S}_{\mathrm{one}-\mathrm{way}}=\underset{\varepsilon_1,{\varepsilon}_2}{\max}\left({p}_{00}+{p}_{10}+{p}_{11}\right)$; (where *X*_1_ depends on *X*_2_).

It is important to mention that the presence of *p*_00_ is required to distinguish *co-presence* patterns from cases when both organisms are simply present in all samples. *Co-presence* patterns require the existence of *co-absence* between the microorganisms in the sample set. Additionally, *co-exclusion* and *one-way* relation pattern scores include *p*_00_ because mutual absence does not contradict the pattern.

While presence/absence threshold optimization allows considering that different microorganisms may have various minimal abundance thresholds to interact with the MC, this approach also can produce misleading results. For example, a perfect *co-presence* score may be achieved by increasing the presence/absence threshold to the point where all the observations will be counted as absent: *ε*_1_ ≥ (*X*_1_)  and *ε*_2_ ≥ (*X*_2_) .

This effect can be minimized by requiring a proportion of experimental observations in quadrants contributing to the pattern under consideration to be above a predefined minimal threshold (*m*):

$$ {S}_{\mathrm{co}-\mathrm{presence}}=\underset{\varepsilon_1,{\varepsilon}_2}{\max}\left({p}_{00}+{p}_{11}\right) $$, where *p*_00_ > *m*; *p*_11_ > *m*;

$$ {S}_{\mathrm{co}-\mathrm{exclusion}}=\underset{\varepsilon_1,{\varepsilon}_2}{\max }\ \left({p}_{00}+{p}_{10}+{p}_{01}\right) $$, where *p*_01_ > *m*; *p*_10_ > *m*;

$$ {S}_{\mathrm{one}-\mathrm{way}}=\underset{\varepsilon_1,{\varepsilon}_2}{\max}\left({p}_{00}+{p}_{01}+{p}_{11}\right) $$; where *p*_01_ > *m*, *p*_11_ > *m*;

(where *X*_2_ depends on *X*_1_);

$$ {S}_{\mathrm{one}-\mathrm{way}}=\underset{\varepsilon_1,{\varepsilon}_2}{\max}\left({p}_{00}+{p}_{10}+{p}_{11}\right) $$, where *p*_10_ > *m*, *p*_11_ > *m*;

(where *X*_1_ depends on *X*_2_).

### Non-trivial multidimensional patterns

The proposed approach can be further extended to identify more complex multidimensional patterns. For example, in some 3D patterns, the presence or absence of one organism may define the kind of 2D patterns exhibited between two other organisms. Figure 1f shows a case where organisms 2 and 3 will be *co-present* if organism 1 is present and *co-exclude* if this organism is absent:

$$ {S}_{\mathrm{pattern}\ \mathrm{A}}=\underset{\varepsilon_1,{\varepsilon}_2,{\varepsilon}_3}{\max }\ \left({p}_{000}+{p}_{111}+{p}_{010}+{p}_{001}\right) $$, where *p*_111_ > *m*; *p*_010_ > *m*; *p*_001_ > *m*.

Similar to 2D patterns, not all combinations of *p*_*ijk*_ values can be interpreted as possible relations between microorganisms. Some 3D patterns can be the direct result of three pairwise 2D patterns: for example, the pairwise *co-exclusion* pattern between three organisms will unambiguously lead to a 3D *co-exclusion* pattern (Fig. 1d):

$$ {S}_{3\mathrm{D}\ \mathrm{co}-\mathrm{exclusion}\ \mathrm{type}\ 1}=\underset{\varepsilon_1,{\varepsilon}_2,{\varepsilon}_3}{\max }\ \left({p}_{000}+{p}_{100}+{p}_{010}+{p}_{001}\right) $$, where *p*_100_ > *m*; *p*_010_ > *m*; *p*_001_ > *m*;

Is a direct consequence of its 2D patterns:

$$ {S}_{\mathrm{co}-\mathrm{exclusion}\ 1,2}=\underset{\varepsilon_1,{\varepsilon}_2}{\max }\ \left({p}_{000}+{p}_{100}+{p}_{010}\right) $$, where *p*_010_ > *m*; *p*_100_ > *m*;

$$ {S}_{\mathrm{co}-\mathrm{exclusion}\ 1,3}=\underset{\varepsilon_1,{\varepsilon}_2}{\max }\ \left({p}_{000}+{p}_{100}+{p}_{001}\right) $$, where *p*_100_ > *m*; *p*_001_ > *m*;

$$ {S}_{\mathrm{co}-\mathrm{exclusion}\ 2,3}=\underset{\varepsilon_1,{\varepsilon}_2}{\max }\ \left({p}_{000}+{p}_{010}+{p}_{001}\right) $$, where *p*_001_ > *m*; *p*_010_ > *m*.

Three-dimensional *co-exclusion* patterns may additionally be observed in a very different way where each pair of organisms is *co-present* only if the third one is absent (Fig. 1e):

$$ {S}_{3\mathrm{D}\ \mathrm{co}-\mathrm{exclusion}\ \mathrm{type}\ 2}=\underset{\varepsilon_1,{\varepsilon}_2,{\varepsilon}_3}{\max }\ \left({p}_{000}+{p}_{110}+{p}_{101}+{p}_{011}\right) $$, where *p*_110_ > *m*; *p*_101_ > *m*; *p*_011_ > *m*.

In fact, every 2D pattern has at least one non-trivial 3D analog which can be interpreted as the relation between organisms and not derived directly from any 2D combination:

$$ {S}_{3\mathrm{D}\ \mathrm{co}-\mathrm{presence}}=\underset{\varepsilon_1,{\varepsilon}_2,{\varepsilon}_3}{\max }\ \left({p}_{000}+{p}_{111}\right) $$, where *p*_111_ > *m*; *p*_000_ > *m*;

$$ {S}_{4\mathrm{D}\ \mathrm{co}-\mathrm{presence}}=\underset{\varepsilon_1,{\varepsilon}_2,{\varepsilon}_3}{\mathit{\max}}\ \left({p}_{0000}+{p}_{1111}\right) $$, where *p*_1111_ > *m*; *p*_0000_ > *m*;

or for one-way relations:

$$ {S}_{3\mathrm{D}\ \mathrm{one}-\mathrm{way}\ \mathrm{relation}}=\underset{\varepsilon_1,{\varepsilon}_2,{\varepsilon}_3}{\max }\ \left({p}_{000}+{p}_{111}+{p}_{001}+{p}_{010}\right), $$

where *p*_111_ > *m*; *p*_001_ > *m*; *p*_010_ > *m*; (organism 1 requires two others to be present).

$$ {S}_{4\mathrm{D}\ \mathrm{one}-\mathrm{way}\ \mathrm{relation}}=\underset{\varepsilon_1,{\varepsilon}_2,{\varepsilon}_3,{\varepsilon}_4}{\max }\ \left({p}_{0000}+{p}_{1111}+{p}_{0001}+{p}_{0010}+{p}_{0100}\right), $$

where *p*_1111_ > *m*; *p*_0001_ > *m*; *p*_0010_ > *m*; *p*_0100_ > *m*;

(organism 1 requires three others to be present).

Additionally, some high dimensional patterns can reflect interesting new relations which exist only in higher dimensions. Figure 1f presents a case where two microorganisms (*X*_1_ and *X*_2_) follow *co-exclusion* patterns in the presence of the third (*X*_3_), as well as *co-presence* in its absence; Fig. 1g shows a case where three organisms can be present only all together or individually:

$$ {S}_{3\mathrm{D}\ \mathrm{all}\ \mathrm{together}\ \mathrm{or}\ \mathrm{alone}}=\underset{\varepsilon_1,{\varepsilon}_2,{\varepsilon}_3}{\max }\ \left({p}_{000}+{p}_{111}+{p}_{001}+{p}_{010}+{p}_{100}\right), $$

where *p*_111_ > *m*; *p*_100_ > *m*; *p*_001_ > *m*; *p*_010_ > *m*; (three organisms present only all together or individually).

### Statistical significance and type 1 error

It is important to keep in mind that an arbitrary choice of the presence threshold (*m*) and minimal score (*S*_min_) above which patterns are considered to be present can significantly affect the results of the analysis in both: a number of detected patterns and their statistical significance (e.g., type 1 error). Lowering these thresholds increases chances for patterns to appear randomly, and this can be detected by comparing the results produced by real data against a randomized (shuffled) dataset. Table 1 provides an example of the number of two-dimensional *one-way* relation patterns identified in original and shuffled (and renormalized) datasets from the Human Microbiome Project (genus level, mid-vagina samples) [22, 23].

**Table 1:** The number of two-dimensional *one-way* relation patterns identified in shuffled and real (original) mid-vagina samples. Bold font reflects the score/threshold combinations where no patterns have been observed in simulated (shuffled) data

Number of patterns in real data/number of patterns in shuffled data											
Population threshold/score	0.90	0.91	0.92	0.93	0.94	0.95	0.96	0.97	0.98	0.99	1
0.05	268/166	257/144	234/124	234/124	220/110	189/78	166/59	135/35	98/19	57/10	57/10
0.075	149/63	140/55	120/46	120/46	104/35	86/26	75/20	62/13	48/4	30/2	30/2
0.1	83/31	78/25	68/21	68/21	65/18	51/10	41/8	35/4	23/2	**15/0**	**15/0**
0.125	54/13	50/10	47/9	47/9	43/6	36/4	31/1	24/1	18/1	**13/0**	**13/0**
0.15	34/8	31/5	27/3	27/3	26/1	21/1	18/1	14/1	12/1	**11/0**	**11/0**
0.175	18/7	16/2	16/1	16/1	15/1	**10/0**	**10/0**	**8/0**	**6/0**	**3/0**	**3/0**
0.2	13/3	12/1	12/1	12/1	**12/0**	**9/0**	**7/0**	**7/0**	**6/0**	**2/0**	**2/0**
0.225	7/1	**7/0**	**7/0**	**7/0**	**6/0**	**5/0**	**4/0**	**4/0**	**4/0**	**2/0**	**2/0**
0.25	**5/0**	**4/0**	**4/0**	**4/0**	**4/0**	**4/0**	**4/0**	**4/0**	**4/0**	**2/0**	**2/0**
0.275	**4/0**	**4/0**	**4/0**	**4/0**	**4/0**	**4/0**	**4/0**	**4/0**	**4/0**	**2/0**	**2/0**
0.3	**4/0**	**4/0**	**4/0**	**4/0**	**4/0**	**4/0**	**4/0**	**4/0**	**4/0**	**1/0**	**1/0**
0.325	**1/0**	**1/0**	**1/0**	**1/0**	**1/0**	**1/0**	**1/0**	**1/0**	**1/0**	0/0	0/0
0.35	0/0	0/0	0/0	0/0	0/0	0/0	0/0	0/0	0/0	0/0	0/0

The choice of the shuffling method reflects the underlying assumption about what would be considered as the random alternative to the observed dataset (zero model) [14, 24]. For example, the shuffling of the abundance values across the whole dataset reflects the assumption of the total randomness of the appearances of all the values across samples and organisms. While this model preserves the overall distribution of the abundance values, it does not take into consideration that some organisms may always be present in low abundance and others can become highly dominated species in the community. In order to reflect this property on microbial abundance data, shuffling across individual OTU profiles has been implemented in the presented method and used in all the examples shown in this manuscript.

The shuffling approach has been implemented as part of all pattern-specific computational pipelines (see the “Methods” section) to make sure that the search for the patterns in real data is performed only for the presence (*m*) and minimal score (*S*_min_) thresholds for which the number of specific patterns in shuffled data is equal to zero. The presented method, however, allows a variety of modifications including less strict type 1 error requirements. The next versions of the software will include the ability to perform multiple shuffling types as well as the ability to perform shuffling multiple times.

### Implementation

The presented method is able to identify three types of 2D patterns (*co-presence*, *co-exclusion*, and *one-way* relations) as well as three types of 3D patterns shown on Fig. 1e–g. The codebase was developed in C++, and the executable files and source code are available on GitHub (https://github.com/kkhanipov/MultidimensionalBooleanPatterns).

In order to improve performance in the proposed implementation, the patterns in shuffled data for all the combinations of presence threshold (*m*) and minimal score (*S*_min_) are calculated during the first step of the analysis, so search for patterns in real data can be performed in a limited search space where zero patterns are detected in randomized (shuffled) data.

To evaluate performance, the presented source code was compiled using a GCC compiler version 6.3.1 under Linux CentOS 6.7. Sixteen HMP OTU files were used for the identification of 2D and 3D patterns on 4× AMD Opteron 8 core processors, 512 GB RAM, and 30 TB of storage system. Search for the two-dimensional *co-presence*, *co-exclusion*, and *one-way relation* patterns for all tested samples took between 1 and 3 min, and the memory footprint did not exceed 50 Mb of RAM. However, search for the 3D patterns may take hundreds of hours and requires a higher level of parallelization and a high-performance computing environment.

### Data acquisition

The microbial community compositions used for this analysis originated from the NIH Human Microbiome Project [22, 23] and contained 18 datasets associated with 16 body sites. Microbial profiles for 2910 samples have been downloaded from the project website as of December 2016 in text format (HMQCP–QIIME community Profiling v13 OTU table). Samples representing significantly low (less than 2000) and significantly high (over 50,000) number of sequencing reads were excluded from the analysis. The microbial profiles of the remaining 2380 samples, varying from 67 for posterior fornix to 200 for antecubital fossa, have been normalized against the total number of reads in each sample and transformed into relative abundance profiles merged to genus taxonomy level for each body site resulting in 619 profiles. Analysis has been performed for each body site individually. For each body site under consideration, genera present in less than 5% of samples have been excluded from the analysis.

### Boolean patterns detected in Human Microbiome Project data

**Fig. 3:**
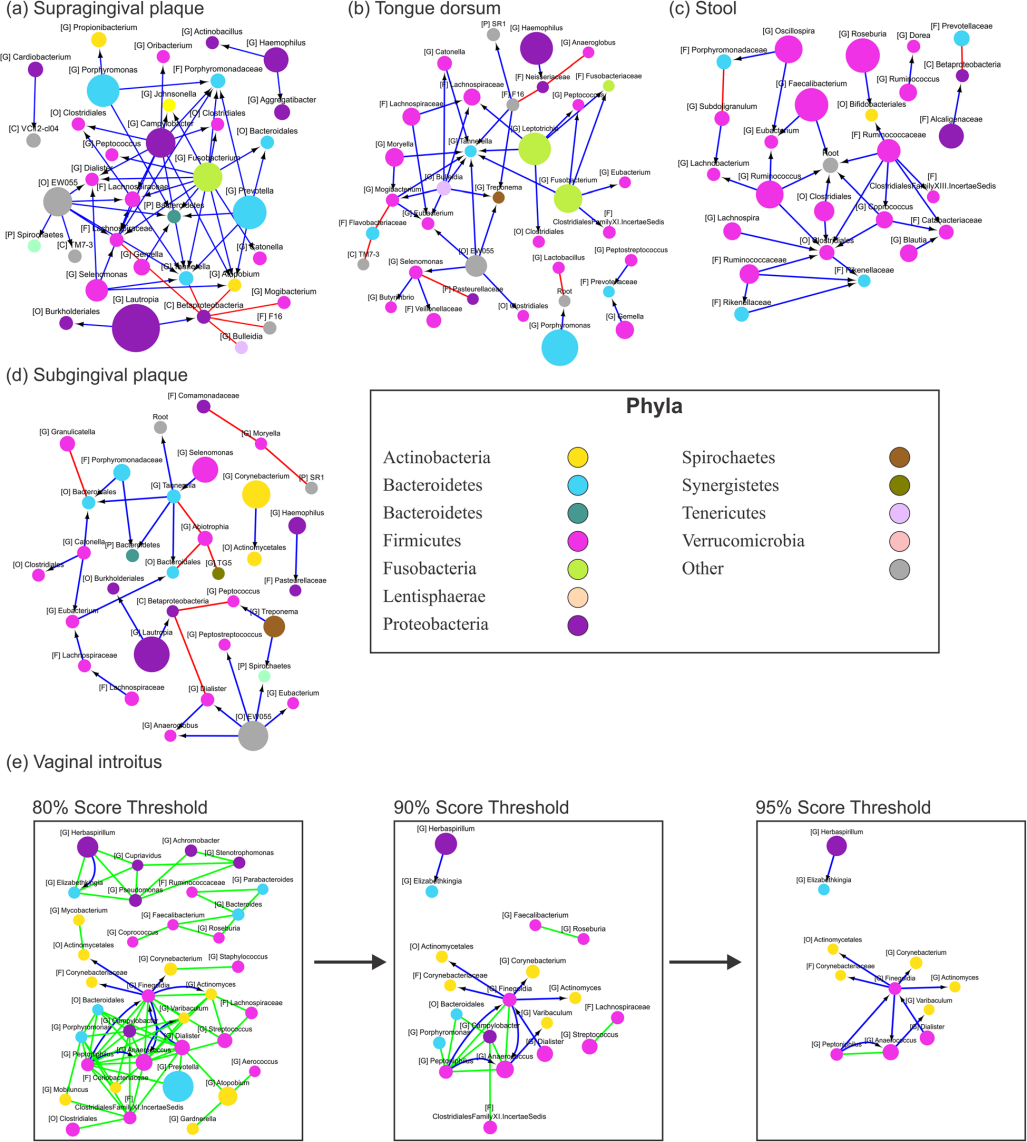
2D microbe-microbe interaction networks. 2D networks for **a** supragingival plaque, **b** tongue dorsum, **c** stool, **d** subgingival plaque, and **e** vaginal introitus samples at the genus level. Example of the effects of the patterns’ score threshold on the network’s complexity (**e**). Node colors reflect different taxonomy assignments at phylum level, and node sizes are proportional to the average relative abundance of the microorganism across samples. Capital letters inside square brackets represent the lowest taxonomy level identified for each OTU: G, genus; F, family; O, order; C, class; and P, phyla. The color of edges indicates relationship type: blue with a black arrow (*one-way* relations), red (*co-exclusion*), and light green (*co-presence*)

All three types of 2D patterns have been identified in virtually every type of sample of the Human Microbiome Project data (Additional File 1). The largest number of patterns (all types included) has been detected in supragingival plaque, tongue dorsum, stool, and subgingival plaque datasets (Fig. 3a–d and Additional File 2). No apparent correlation has been observed between the number of patterns and the total number of samples nor the number of OTUs in the datasets. It is important to mention that while all of the observed 2D patterns pass statistical significance criteria, the overall size and complexity of the resulting networks depend on the pattern score threshold (Fig. 3e). In the interaction patterns of HMP supragingival (Fig. 3a), the *Fusobacteria* genus (green) node has 11 one-way relationships with other taxa. Additionally, there are another 12 such significant patterns with lower scores as shown in the supplementary file “Additional File 1.” *Fusobacteria nucleatum* is a well-known pathogen that was not only found in the subgingival and supragingival plaques [25], but also previously characterized in vitro [26] and in vivo [27] dental plaque biofilms. Interestingly, one group found out that *F. nucleatum* is unable to grow as a single species and builds a mutualistic relationship with other members of local microbiota such as *Aggregatibacter.* The presence of this pattern is in agreement with previous findings that orofacial odontogenic infections are usually polymicrobial [28]. While the *Fusobacterium*-*Aggregatibacter* pattern has not been identified in our cohort of samples, our findings of one-way relation patterns between *Fusobacterium* and other members of microbial communities yield rather interesting results. Thirteen such patterns showed interaction between *Fusobacteria* spp. and other known pathogens. For example, *Catonella* and *Clostridiales* spp. have been previously associated as uniquely present in the patients with caries [29]. Another pattern includes a one-way relation with *Tannerella* spp., which is also known as periodontal pathogen [30], as well as very well-studied *Dialister* spp. and their role as periodontopathic bacteria [31]. Finally, while species of identified *Johnsonella* genus are not known directly related to dental diseases, they have been linked to chronic obstructive pulmonary disease (COPD) which strengthen proposed method as a tool for hypothesis discovery in microbial communities [32].

While the available HMP data does not possess enough precision power to pinpoint the exact pathogenic strains that are contained within the samples and which happen to be within the pathogenic genera, family, or even order, we believe these observations of interaction between *Fusobacteria* and other members of microbial communities are not random and may benefit from further analysis and validation. Improvement of high-throughput sequence technologies, decrease of cost, and availability of the high-quality public data will close this data precision gap.

Some 3D patterns have also been observed in buccal mucosa, supragingival plaque, and merged retroauricular crease datasets (see example in Table 2).

**Table 2:** Example of 3D patterns where organisms 2 and 3 are *co-present* if organism 1 is present and *co-excluded* if organism 1 is absent identified in anterior nares samples (genus level). Calculations have been performed with the minimum population threshold set to 0.1 Capital letters inside square brackets represent the lowest taxonomy level identified for each OTU: *G* genus, *F* family, *O* order, *C* class, and *P* phyla

Organism 1	Organism 2	Organism 3	Pattern score	*p*_000_	*p*_001_	*p*_010_	*p*_011_	*p*_100_	*p*_101_	*p*_110_	*p*_111_
[G] *Actinomyces*	[G] *Rothia*	[G] *Neisseria*	0.94	0.48	0.12	0.18	0.05	0.00	0.00	0.02	0.16
[F] Ruminococcaceae	[G] *Bacteroides*	[F] Lachnospiraceae	0.94	0.54	0.12	0.17	0.03	0.02	0.00	0.00	0.11
[G] *Faecalibacterium*	[G] *Bacteroides*	[F] Lachnospiraceae	0.94	0.57	0.12	0.16	0.04	0.00	0.00	0.02	0.10
[G] *Oscillospira*	[G] *Bacteroides*	[F] Lachnospiraceae	0.94	0.60	0.12	0.12	0.03	0.01	0.01	0.02	0.10
[G] *Oscillospira*	[F] Lachnospiraceae	[G] *Faecalibacterium*	0.94	0.57	0.10	0.17	0.04	0.01	0.02	0.00	0.10
[G] *Neisseria*	[G] *Rothia*	[G] *Prevotella*	0.94	0.56	0.15	0.12	0.02	0.01	0.01	0.02	0.11
[G] *Neisseria*	[G] *Rothia*	[G] *Veillonella*	0.94	0.48	0.23	0.10	0.04	0.01	0.01	0.00	0.13

## Discussion and conclusion

Identification of interaction patterns in microbial communities is essential to further our understanding of relationships in microbial communities. Knowledge of the interactions between specific organisms can help transition microbiomes between enterotypes and better predict microbial responses due to perturbations (e.g., targeted antimicrobials, probiotics, prebiotics). Ability to manipulate microbial communities in terms of community members and their functions will open new opportunities for precision medicine and personalized treatments. Thus, the development of systematic and statistically sound methods for interaction pattern identification is a necessary step to understand the structure of microbiomes and the processes by which they evolve.

Since correlation and MI-based approaches can miss important multidimensional patterns and produce misleadingly low scores for certain intuitively obvious patterns, the proposed method could serve as a useful addition to the set of tools available for the microbiome analysis. It is important to keep in mind, however, that the presence of statistically significant patterns between the abundance of two and more organisms must be interpreted very carefully and treated more like an indication of a potential interaction and requires independent experimental validation. Additionally, datasets from different environments or conditions should not be analyzed simultaneously for patterns, since this may result in false interaction patterns such as co-exclusion due to the datasets being of different nature with different compositions. The comparison of interactions between different conditions should be done between the calculated interaction pattern sets (networks).

**Fig. 4:**
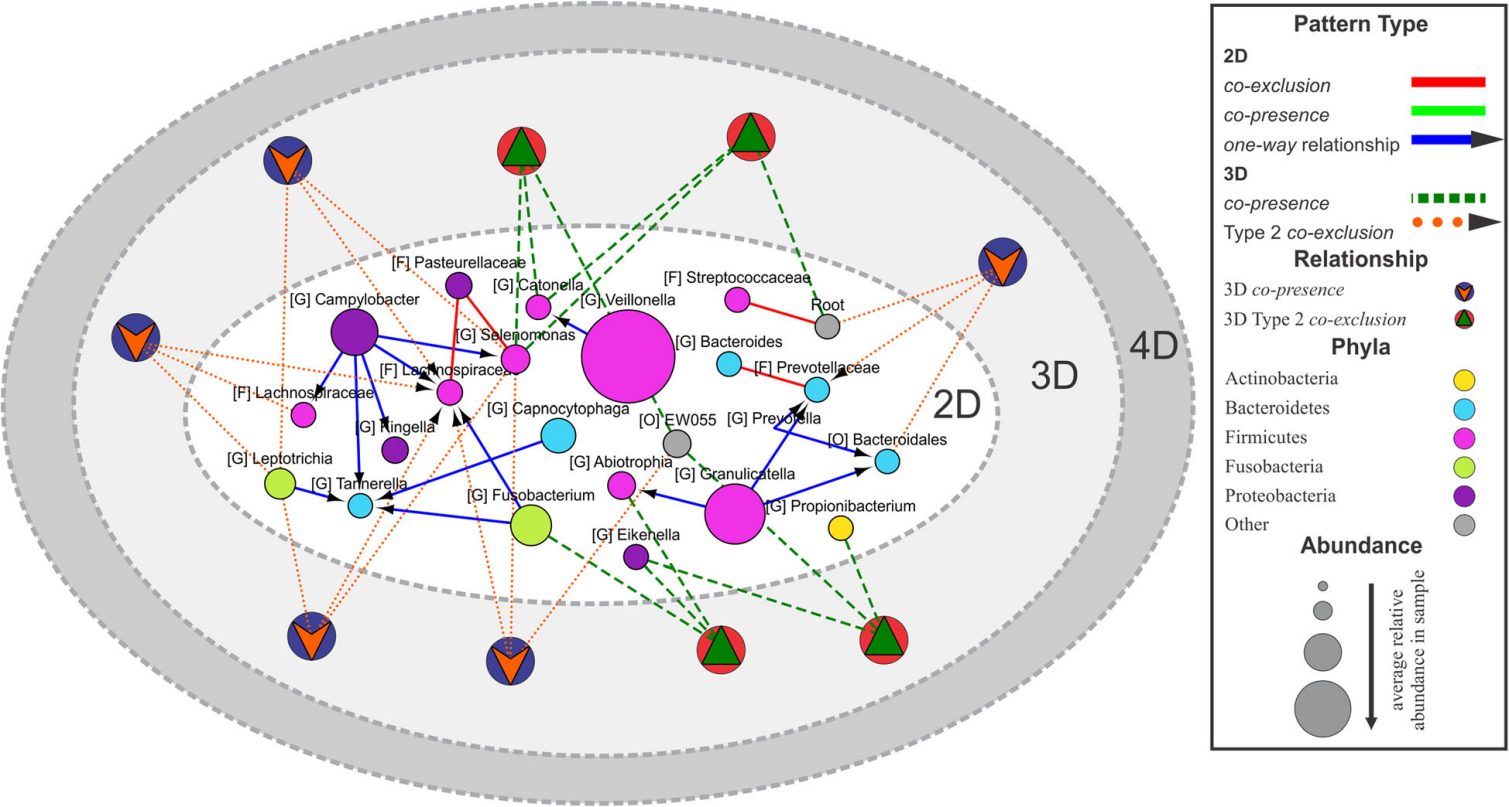
Example of multi-layer network. Multi-layer network visualization of two- and three-dimensional patterns in attached keratinized gingiva samples. The network contains two types of nodes representing OTUs (circular) and three-dimensional patterns (circular with a triangle). Node colors reflect different taxonomy assignments at phylum level, and node sizes are proportional to the average relative abundance of the microorganism across samples. Capital letters inside square brackets represent the lowest taxonomy level identified for each OTU: G, genus; F, family; O, order; C, class; and P, phyla. The color of edges indicates relationship type: blue with a black arrow (*one-way* relations), red (*co-exclusion*), light green (*co-presence*), dark green (3D *co-presence*), and orange (type 2 *co-exclusion*)

The visualization of multidimensional patterns involving multiple organisms, however, remains a significant challenge. Traditionally, the graph (network) representation of the patterns between organisms in microbial communities represents each OTU as the node and pairwise relationships as edges [14, 16, 24, 33]. We believe that one of the possible ways to visualize 2D, 3D, and higher dimensional patterns could be by using a multi-layer network (multi-layer graph) which in contrast with traditional graphs (networks) can simultaneously include nodes of different types [34], such as OTUs and multidimensional patterns. Figure 4 shows an example of such a representation for two- and three-dimensional patterns in attached keratinized gingiva samples from the Human Microbiome Project.

The presented approach can also be extended by including a variety of physical (pH, temperature, oxygen concentration) and biochemical (antimicrobial susceptibility, nutrient, and metabolite concentration) variables into the search for multidimensional patterns. We also believe that it can be extended to the simultaneous analysis of multi-omics data, such as protein and mRNA expression in both microbial communities and the mammalian host.

## Supplementary information

**Additional file 1.** 2 Dimensional Patterns. 2D Patterns generated from Human Microbiome Project by Body Site.

**Additional file 2.** 2 Dimensional Networks. 2D Network generated from Human Microbiome Project by Body Site.

## Data Availability

The datasets analyzed during the current study are available in the Human Microbiome Project repository (https://portal.hmpdacc.org/) [35]. All data generated or analyzed during this study are included in this published article and its supplementary information files. Executable files and source code are available on GitHub (https://github.com/kkhanipov/MultidimensionalBooleanPatterns).
